# Improvements in clinical response between 12 and 24 weeks in patients with rheumatoid arthritis on etanercept therapy with or without methotrexate

**DOI:** 10.1136/ard.2008.094524

**Published:** 2008-06-05

**Authors:** A Kavanaugh, L Klareskog, D van der Heijde, J Li, B Freundlich, M Hooper

**Affiliations:** 1 Center for Innovative Therapy, Division of Rheumatology, Allergy, Immunology, UCSD, San Diego, California, USA; 2 Rheumatology Unit, Department of Medicine, Karolinska Institutet, Stockholm, Sweden; 3 Department of Rheumatology, Leiden University Medical Center, Leiden, The Netherlands; 4 Amgen Inc, Thousand Oaks, California, USA; 5 Wyeth Pharmaceuticals, Collegeville, Pennsylvania, USA

## Abstract

**Background:**

Whereas many patients respond quickly to treatment with tumour necrosis factor (TNF) inhibitors, some patients may experience significant but delayed responses.

**Objective:**

To evaluate the clinical response between 12 and 24 weeks in subjects with rheumatoid arthritis from the Trial of Etanercept and Methotrexate with Radiographic Patient Outcomes.

**Methods:**

Clinical response was assessed at 24 weeks in 12-week non-responders, according to American College of Rheumatology (ACR) response criteria. The proportion of subjects who successfully maintained response to 52 weeks was analysed, as were radiographic outcomes.

**Results:**

Data from 682 subjects were included in the analysis. Non and partial responders in all three groups (etanercept, methotrexate and etanercept plus methotrexate) at week 12 showed an improvement in responses at week 24. Over 80% of the week 24 ACR20/50/70 responders in the etanercept plus methotrexate arm sustained their response to 52 weeks. In the etanercept arms, a delayed clinical response was not associated with increased radiographic progression at week 52.

**Conclusion:**

A significant proportion of non and partial responders to etanercept with or without methotrexate therapy at week 12 achieved a good clinical response or improved their overall clinical response at week 24. Discontinuing TNF inhibitor therapy at 12 weeks may be premature in some rheumatoid arthritis patients.

Tumour necrosis factor (TNF) inhibitors, particularly in combination with methotrexate, have demonstrated excellent symptomatic and radiographic control in rheumatoid arthritis.^1^ ^2^ A rapid clinical response to such therapy, often within 2 weeks, is commonly observed.^1^ ^3^ ^4^ It is widely held that most patients who respond to TNF inhibitor therapy will show an adequate response after 12 weeks of treatment. Given the cost and possible unnecessary exposure to an ineffective medication, 12 weeks has been suggested as a timepoint at which TNF inhibitor therapy should be discontinued in non-responders. Discontinuing therapy may be premature, however, if a proportion of non-responders at 12 weeks become responders at later timepoints.^5^ ^6^

In this analysis, we used data from the Trial of Etanercept and Methotrexate with Radiographic Patient Outcomes (TEMPO) to evaluate the extent to which subjects not responding to TNF inhibitor therapy at 12 weeks might respond at 24 weeks. The long-term sustainability of the improvement in response and radiographic outcome at week 52 was also assessed.

## SUBJECTS AND METHODS

### Subjects

Data from subjects treated with etanercept, methotrexate and etanercept plus methotrexate from the TEMPO study were used in this analysis.^1^ Briefly, subjects had disease duration of between 6 months and 20 years, had active rheumatoid arthritis defined as 10 or more swollen and 12 or more painful joints and had at least one of the following: erythrocyte sedimentation rate 28 mm/h or greater; C-reactive protein 20 mg/l or greater; or morning stiffness for 45 minutes or more. Etanercept was given as 25 mg twice a week. Patients randomly assigned to methotrexate arms received 7.5 mg methotrexate once a week, which was escalated to 20 mg once a week over 8 weeks.

### Evaluations

American College of Rheumatology (ACR)20/50/70 responses and Disease Activity Score using 28 joints (DAS28) were assessed at weeks 12, 24 and 52. Based on their clinical response at week 12, subjects were categorised into ACR20 non-responders (no response at week 12), ACR50 non-responders (ACR20 responders, but not ACR50 responders) and ACR70 non-responders (ACR50 responders, but not ACR70 responders).

Among the subjects who showed an improvement in clinical response at week 24, the proportion of subjects who successfully maintained the improvement to at least 52 weeks was assessed. The radiographic outcome at week 52 was evaluated using the mean change in total Sharp score (TSS) and the percentage of non-progressors (TSS change ≤0).

Frequencies and percentages were provided for improvement and decrease in ACR response and disease activity scores. One-way analysis of variance was used to test the difference in DAS28 scores among groups (non-responder to non-responder, non-responder to responder and responder to responder). The paired-sample t test was used to examine the difference in DAS28 scores between week 12 and week 24 and for week 12 European League Against Rheumatism (EULAR) non-responders who responded at week 24. Fisher’s exact test was used to compare the difference in radiographic progression between the week 12 responders and week 12 non-responders who became responders at week 24 within each treatment group. Bonferroni adjustment was used for multiple comparisons. Logistic regression models were used to identify predictors of response at 12 weeks. Results were considered significant at p⩽0.05 two-sided. Non-responder imputation was used to handle missing clinical and radiographic data. All analyses were performed using the SAS STAT system version 9.1.

## RESULTS

### Baseline demographics

The TEMPO trial included 682 subjects (231 in the etanercept plus methotrexate arm, 223 in the etanercept monotherapy arm and 228 in the methotrexate monotherapy arm). Baseline demographic characteristics for TEMPO subjects have been described previously.^1^ Briefly, the mean disease duration was 6.8 years, with 76% of subjects rheumatoid factor positive. The mean age was 52.5 years and approximately three-quarters were women. Subjects had a mean baseline DAS28 score of 6.8.

### Improvement in ACR response

**Figure 1:**
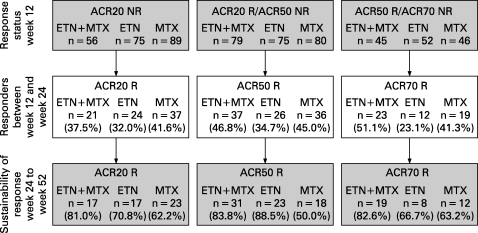
Improvement and sustainability of American College of Rheumatology response.  ACR, American College of Rheumatology; ETN, etanercept; MTX, methotrexate; N, total of subgroup; NR, non-responder; R, responder.

Results from the analysis of ACR20/50/70 responses at week 24 in week 12 non and partial responders are shown in fig 1. In the etanercept plus methotrexate-treated subjects, 37.5% of week 12 ACR20 non-responders became ACR20 responders, 46.8% of week 12 ACR50 non-responders became ACR50 responders and 51.1% of week 12 ACR70 non-responders became ACR70 responders. Similar improvements were also observed in the monotherapy arms (23%–35% in the etanercept monotherapy arm and 41%–45% in the methotrexate monotherapy arm).

### Sustainability of ACR response

Of 21 ACR20, 37 ACR50 and 23 ACR70 responders at week 24 in the etanercept plus methotrexate arm, 17 (81.0%), 31 (83.8%) and 19 (82.6%) subjects, respectively, showed a sustained response at week 52 (fig 1). The sustainability of response was more variable in the etanercept and methotrexate monotherapy arms, ranging from 67% to 89% and 50% to 63%, respectively.

A decrease in ACR20/50/70 response was observed in some subjects between weeks 12 and 24. In the etanercept plus methotrexate arm, 18 out of 175 ACR20 responders (10.3%), five out of 96 ACR50 responders (5.2%) and five out of 51 ACR70 responders (9.8%) decreased their response at week 24 (data not shown). Similarly, 11.5% of ACR20, 23.3% of ACR50 and 4.8% of ACR70 responders in the etanercept monotherapy arm and 16.5% of ACR20, 10.2% of ACR50 and 23.1% of ACR70 responders in the methotrexate monotherapy arm decreased their response between weeks 12 and 24.

### Improvement in DAS28 scores

**Figure 2:**
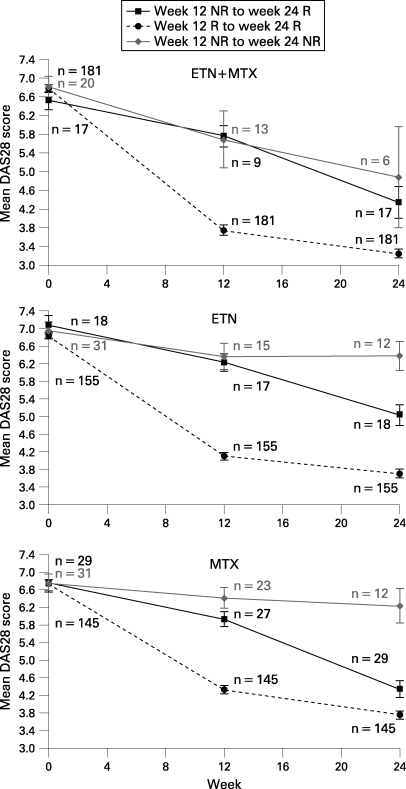
Mean DAS28 scores in EULAR (moderate or good) responders and non-responders.  DAS28, Disease Activity Score using 28 joints; ETN, etanercept; EULAR, European League Against Rheumatism; MTX, methotrexate; N, total of subgroup; NR, non-responder; R, responder.

In subjects from all three treatment arms who were EULAR (moderate or good) non-responders at week 12, but became responders at week 24, their mean DAS28 scores showed a significant decrease from baseline to week 24 and from week 12 to week 24 (p<0.05) (fig 2). In the non-responder to non-responder group, mean DAS28 scores at week 12 were similar to those in the non-responder to responder group, arguing against a “partial response” as the explanation for the later improvement for those patients. Of note, mean DAS28 scores in the 12-week non-responders/24-week responders improved significantly at 24 weeks, but not to the same extent as for subjects who achieved a response at 12 weeks.

### Radiographic outcome at week 52

To determine if there was a slowing of radiographic progression in subjects who were ACR20 non-responders at week 12, but became responders at week 24 compared with week 12 responders, the mean change in TSS and the percentage of non-progressors were evaluated at week 52. Results are shown in table 1. No statistically significant difference was observed in the mean TSS or the percentage of non-progressors between the 12-week versus the 24-week responders in the etanercept or etanercept plus methotrexate arms. In the methotrexate arm, however, the 24-week responders showed a significant increase in TSS at 52 weeks compared with the 12-week responders.

**Table 1:** Radiographic outcome at week 52 in week 12 and week 24 responders

	N	NP (%)	p Value	Mean TSS (95% CI)
ETN + MTX week 12 responder	171	69.0	0.440	−0.53 (−0.880 to −0.185)
ETN + MTX week 24 responder	20	80.0		−1.35 (−2.472 to −0.234)
ETN week 12 responder	145	59.3	0.177	−0.23 (−0.690 to 0.216)
ETN week 24 responder	24	75.0		−0.19 (−1.043 to 0.662)
MTX week 12 responder	133	48.1	0.037	0.64 (0.078 to 1.202)
MTX week 24 responder	36	27.8		2.82 (−0.487 to 6.145)

ETN, etanercept; MTX, methotrexate; NP, non-progressor defined by TSS ⩽0; TSS, total Sharp score.

### Predictors of improvement in response

No baseline characteristics or week 12 clinical variables (C-reactive protein, erythrocyte sedimentation rate, swollen joint count, tender joint count, Health Assessment Questionnaire, visual analogue scale for pain, physician global assessment and DAS28) were significantly associated with a delayed response at 24 weeks.

## DISCUSSION

In this retrospective analysis, a substantial improvement in clinical response was observed between 12 and 24 weeks in subjects with rheumatoid arthritis in all three treatment arms from the TEMPO trial, including those who were non or partial responders at 12 weeks. The improvement in response at 24 weeks was most apparent in the etanercept plus methotrexate arm, in which 37.5% of ACR20, 46.8% of ACR50 and 51.1% of ACR70 non-responders became ACR20, ACR50 and ACR70 responders, respectively, at week 24. Similar improvements were observed for DAS28 scores in all three treatment arms.

The delayed response observed at 24 weeks was largely sustained in 80% of subjects to 52 weeks and was not associated with an inferior outcome with regard to the inhibition of radiographic progression at 52 weeks in the etanercept and etanercept plus methotrexate arms.

There are no clear guidelines on the appropriate treatment trial duration for TNF inhibitors. Recommendations from international guidelines vary, with most indicating 12 weeks as a time to assess response after TNF inhibitor therapy. Whereas some recommend discontinuing therapy at 12 weeks for non-responders, others allow for doctor’s discretion on whether or not to continue therapy.^7^^–^12

Importantly, an analysis of disease variables and patient characteristics failed to identify any predictive factors.

The implications of this assessment are relevant with regard to the subsequent treatment of patients “failing” TNF inhibitor therapy at 12 weeks. It has been shown that patients who have “failed” therapy with a TNF inhibitor have somewhat lesser ACR responses to any second-line biological therapy.^13^^–^15 Therefore, optimising the first treatment course with a TNF inhibitor could improve primary response rates and result in less switching between biological agents.

Limitations of the current study include the retrospective nature of the analysis. Moreover, these findings are from patients enrolled in a randomised clinical trial who met the specific inclusion criteria and had high disease activity. Therefore, these observations may not reflect patients in a real-world setting.

In conclusion, findings from this retrospective analysis suggest that a significant proportion of patients who do not achieve a clinical response 12 weeks after the initiation of etanercept with or without methotrexate therapy may benefit from continuing therapy for up to 24 weeks. Additional benefits of continuing therapy for up to 24 weeks include the sustainability of the 24-week gain in response through at least one year while maintaining radiographic protection.
